# Dying in residential care homes during the early COVID-19 pandemic: a qualitative interview study

**DOI:** 10.1186/s12877-025-05779-y

**Published:** 2025-02-24

**Authors:** Nancy Preston, Zoë Cockshott, Siân Russell, Rachel Stocker, Jo Knight, Suzanne Mason, Barbara Hanratty

**Affiliations:** 1 International Observatory on End of Life Care, Faculty of Health and Medicine, Lancaster University, Lancaster, UK; 2 Population Health Sciences Institute, Faculty of Medical Sciences, Newcastle University, Newcastle, UK; 3 School of Biomedical, Nutritional and Sport Sciences, Faculty of Medical Sciences, Newcastle University, Newcastle, UK; 4 Lancaster Medical School, Faculty of Health and Medicine, Lancaster University, Lancaster, UK; 5 Sheffield Centre for Health and Related Research, School of Medicine and Population Health, University of Sheffield, Sheffield, UK

**Keywords:** Care homes, Long-term care facilities, Palliative care, End-of-life care, COVID-19, Nursing homes, Pandemic

## Abstract

**Background:**

Early in the COVID-19 pandemic, care homes (long-term care facilities) globally were severely impacted in many ways, including end-of-life care and death of residents. They experienced significantly elevated mortality rates amongst residents, compounded by restrictions on support from external healthcare and specialist palliative care providers. Family access to dying residents was often severely restricted. This paper explores experiences of deaths, dying and end-of-life care in care homes during the first year of the pandemic (Spring 2020–2021).

**Methods:**

As part of a wider study of experiences in care homes in Northern England during the early pandemic, we conducted semi-structured interviews with care home staff (16), residents (3), family members (5) and health service staff (10). Interviews were analysed using reflexive thematic analysis, this secondary analysis focusing on experiences of death and dying over the period.

**Results:**

Thematic analysis generated three key themes: *(1) Preparing for large scale deaths*: Care home staff reported a sense of foreboding at requirements to prepare for large scale resident deaths, sometimes feeling left with minimal external support to manage this, and uneasy about the rapid roll-out of emergency care planning to residents; *(2) Balancing support and policing visiting during the terminal phase*: The requirement to restrict access for family members when their relatives were dying was experienced as distressing for both family members and care home staff; and, (3) *Distress surrounding deaths for staff and families*: Care home staff were distressed by the frequency and speed of deaths that they witnessed when their care home had a COVID-19 outbreak. Family separation near time of death was a source of distress for everyone involved, with suggestions that this led to regrets in bereavement for family members, and moral distress in staff.

**Conclusions:**

The experience of death and dying in care homes in the early waves of the COVID-19 pandemic was extremely challenging for care home staff and family members. Our analysis suggests that the ramifications of stringent visitation policies and the consequent distress may shape experiences in bereavement. Monitoring for longer term consequences, such as prolonged grief and moral injury, should be a priority.

**Supplementary Information:**

The online version contains supplementary material available at 10.1186/s12877-025-05779-y.

## Introduction

Care homes^1^ (also known internationally as residential long term care facilities and residential aged care facilities) experienced particularly high mortality rates during the early waves of the COVID-19 pandemic in 2020/2021. Precise figures on COVID-19-related deaths in these settings at the time are unclear due to early challenges in virus testing. However, estimates suggest that care home resident deaths accounted for up to 41% of COVID-19 deaths in 2020 both in the UK [1] and internationally [2]. In several countries, including the UK and the USA, more than 5% of care home residents died during the early waves of the pandemic [3].

In many countries, staff in care homes were required to adapt their practices at pace, to respond to the threat posed by the COVID-19 virus, and to changes in government guidance [4–6]. Care homes were subject to prolonged lockdowns, and as the pandemic progressed, restricted access to them continued [7–9]. These restrictions were associated with a steep fall in the number of visitors, including external clinical staff, friends and family [10], and reduced access to external services including attendance at emergency departments, hospital admission and community appointments [11, 12]. These visiting restrictions were also in place when residents were dying, meaning that residents and their families were largely separated at the end-of-life [10, 13, 14, 15]. In addition, concerns were raised early in the pandemic regarding the blanket application of Do Not Attempt Resuscitation (DNAR) orders to care home residents with little or no consultation with residents or family [16–18]. These factors, along with a substantially increased number of deaths in care homes, suggest that the experiences of dying and end-of-life care in care homes were considerably altered. This study set out to understand more about these experiences.

Prior to the pandemic, the nature of care home work presented a challenging picture, where a traditionally low-paid, and often under-recognised workforce provides care to a frequently vulnerable population with complex health needs [4, 19]. The provision of end-of-life care in care homes has also been described as complex [20], with research identifying a need for improved collaboration between health care teams and care homes, and clarification of the roles and responsibilities of care home staff at the end of life [20]. In addition, Marcella et al. 2015, described frequent difficulties in the way that care home staff were affected by the death of residents [21]. Whilst Marcella et al. [21] found that the death of residents was considered ‘part of the job’ *(p1)*, deaths were often hidden away and not discussed openly amongst care home staff. What were seen as ‘good deaths’ were less emotionally affecting than ‘bad deaths’, which were characterised by pain and dying alone. These issues of dying apart from family and limited social contact at the end of life, came to the fore in the early pandemic.

Studies in palliative care settings during early waves of the COVID-19 pandemic, indicate that staff frequently found the imposition of social distancing restrictions at the end-of-life at odds with their professional values of good care, resulting in ‘moral distress’ [21–24]. Requirements to avoid physical contact, and the use of PPE, were seen as additional barriers to usual communication and care [25]. Studies involving care home staff indicate that provision of quality end-of-life care during the restrictions of the early pandemic was a significant concern for them [9, 13, 14, 26, 27]. Studies involving care home residents’ family members who were bereaved at this time, suggest that lack of access and communication, and lack of physical contact and the ability to say goodbye at the end-of-life were key sources of distress [10, 14, 28]. Bradshaw et al. [29] note that the relationship-centred care which is critical to quality of end-of-life care in care homes was disrupted at this time, including in terms of access to external palliative care support services for some care homes.

Both before, and during, the early waves of the pandemic, care homes struggled to recruit staff to meet the increasingly complex needs of residents [19, 30]. This, together with reductions in external healthcare support due to COVID-19 restrictions [6, 15, 31], resulted in an increase in workload for care staff. In this context, care homes supported residents at the end of their lives, many of whom would have been well known to the care home staff, as would their families [32].

How people died in care homes amidst the challenges of the pandemic, and the impact of this on care home staff residents and family members has had limited research attention. Hence, we wished to explore this experience from interviews conducted at the time, in order to make recommendations for future crises including pandemics. Our work aimed to build on and add to the findings of previous authors [10, 13, 14, 28] regarding end-of-life care in care homes during early waves of the pandemic. In order to achieve this, we focussed on rich and in-depth accounts of dying in care homes from care home and health service staff, residents’ family members, as well as attempting to capture the accounts of residents themselves.

### Study aims

This paper has been developed from a secondary analysis of findings from a wider study that (a) evaluated the implementation of Digital Care Homes Service^2^ application, and (b) explored the experiences of care home staff, residents, families and community nurses who supported them during the early waves of the COVID-19 pandemic (March 2020-June 2021). We have already produced papers on the emotional impact on care home staff during this time [26], and on the implementation of the Digital Care Homes Service application. [11, 31, 33] Here the focus is specifically on findings from qualitative interview data regarding experiences of dying and end-of-life care in care homes during these early waves of the pandemic, as this was identified as a significant issue in our original analyses, warranting further exploration. Specifically, this paper aims to

- Build on the work of other authors [10, 13, 14, 29] exploring the impact of the early pandemic on provision of end-of-life care in care homes.
- Explore the moral and emotional impact of deaths and disrupted provision of end-of-life care on care home staff and bereaved family members.

## Methods

Semi-structured interviews were conducted with 34 participants as part of the wider study outlined above. Participants were care home staff (16) (managers, care staff, nursing staff), care home residents (3), family members (5), and health service staff working closely with care homes (10) (community nursing staff and the Digital Care Homes Service team). Care home staff and residents were from eight care homes in an area of the North of England served by the Digital Care Homes Service, and health service staff were from teams working with care homes in this area. Family member participants were relatives of care home residents in the same area, but due to recruitment limitations were not family of residents in these eight care homes. For this secondary analysis, we focused on data specifically relating to death and dying in care homes, as this was noted as a significant topic of interest during the original analysis.

### Recruitment

Care home staff, residents and health service staff were recruited via initial contact from the Digital Care Homes Service manager, who sent out emails to community health service teams and to care home managers for them to share details of the study with staff and residents in Autumn 2020 and Spring 2021. Opportunities to recruit family members of care home residents through care home managers were limited by COVID-19 restrictions so these participants were recruited via social media through a Facebook advertisement to a local area group shared in June-July 2021. In all cases, respondents to recruitment calls received further information from the research team and informed consent was obtained either electronically, or verbally and audio-recorded in line with Health Research Authority (HRA) guidance.

### Data collection

Semi-structured interviews were conducted between November 2020 and July 2021 by the three researchers (RS, SR, ZC). All were female Research Associates; RS has a doctorate in health psychology, ZC a doctorate in health research, and SR in medical sociology; all with several years’ experience in qualitative research, including extensive experience in conducting semi-structured interviews. SR and RS have experience conducting research involving care homes. while ZC’s research background has focused on palliative care. Due to COVID-19 restrictions, interviews were conducted remotely, either online via Microsoft Teams, Zoom, or by telephone, depending on participants’ preferences. Remote interviewing, which was already becoming more widely used, became commonplace and highly valued during the pandemic [34, 35]. The interview schedule included questions regarding the impact of COVID-19 on the care homes, their staff, residents, and residents’ families (considering the period from the first wave of the pandemic to the time of interview, during the second wave Autumn 2020 – Spring 2021), as well as questions about use of the Digital Care Home Service. Questions regarding the impact of COVID-19 were open-ended and designed to explore the impact of visiting restrictions and infection control requirements, of COVID-19 cases and deaths, and changes in access to health and palliative care services. The interview schedule was developed from one used in an earlier study [36] by members of the research team (BH, SR, RS). It was discussed with a Public Involvement group (including members of the public with an interest in care homes and social care, through experience as a carer or having worked in the sector) in June 2020, prior to interviews. Following this discussion, modified interview schedules were used for each of the groups of respondents (i.e. for care home and health service staff, residents and relatives), for example with relatives being asked fewer questions about the technicalities of health care provision. The interview topic guides are appended in Supplementary Materials (Additional file 1). To avoid distress, residents and relatives were not directly asked questions about death and dying during the period. They were however asked about their opinions and experiences during the COVID pandemic, how it had changed life in, and access to the care home, and the care that residents received. If residents or relatives raised issues about death and dying these were followed up with further questions where appropriate.

In recruitment information and at the start of interviews, participants were informed that they were free to decline any questions and/or take a break from the interview, should they find any questions distressing. Sources of support were also listed on the participant information sheet should participants wish to access support related to what they had discussed during interview. Interviews were transcribed verbatim prior to analysis.

### Analysis

This was a secondary analysis of qualitative interview data from the original wider study described above. The original analysis of the interview data set for the wider study followed the principles of reflexive thematic analysis (RTA) [37, 38]. This was led by SR, RS, and ZC, with additional input from NP and BH, and drew on the guidelines of Braun and Clark’s [37, 38] six-phase framework, *(familiarisation with data*,* generating initial codes; searching for themes; reviewing themes; defining themes and writing up analysis).* A selection of early transcripts from a range of participants were each coded by two or all three of the researchers, to allow for comparison, discussion and development of coding approaches. We have reported the process and the findings of this original analysis elsewhere [26, 33].

The secondary analysis reported in this paper focused on data specifically relating to death, dying and end-of-life care in care homes. The codes and themes from the original analysis were reviewed by ZC and NP, to explore where data relating to this subject had been previously coded, or identified as a theme or sub-theme. ZC and NP collaborated on reviewing these coded data and revisiting original transcripts for additional context and other more general themes relevant to experiences of death and dying in care homes during the pandemic period. In developing codes and themes from both coded and original transcripts, ZC and NP again used reflexive thematic analysis (RTA) as described above, with further input from SR, RS and BH particularly in reviewing and defining themes, and writing up analysis.

ZC, RS, and SR are experienced in conducting qualitative research in the context of ageing, health and illness, and long-term care. NP is a Professor of Palliative Care, and BH a Professor of Primary Care and Public Health. Between them they have extensive experience conducting and leading research on ageing, end-of-life and palliative care, social care and long-term care facilities. A key principle of RTA is that the researchers’ subjectivity will always play a role is shaping the research. As Braun and Clarke (2023) [39] state themselves the research ‘*will always be infused with their subjectivity*,* and they are never a neutral conduit*’ *(p4)*. The value of this ‘*infusion*’ of subjectivity is the experience, existing knowledge, and expertise NP and ZC brought to this analysis, bolstered further by the input from BH, RS, and SR, who reviewed iterations of the developing themes as the paper for the manuscript progressed. Braun and Clarke (2024) propose the use of Reflexive Thematic Analysis Reporting Guidelines (RTARG) [40], along with eight recommendations to enhance methodological congruence [41] in conducting and reporting studies using RTA. They note that these guidelines are more aligned with the values of RTA, which acknowledges the role of subjectivity and a value-based approach to analysis, than procedural checklists such as the Consolidated Criteria for Reporting Qualitative Research (COREQ) guidelines [42]. We are confident that we have used and reported the methodology with rigour and congruence, using the RTARG and Braun and Clark’s [40, 41] recommendations as a guide.

## Results

A total of 28 interviews were conducted with 34 participants: sixteen care home staff from eight care homes, three care home residents, five family members and ten health service staff who worked closely with care homes. Details of the roles and settings of individual participants are shown in Table 1. Details of the eight participating care homes are shown in Table 2. Twenty-four interviews were one-to-one, four were in pairs or small groups (participants’ choice). All were conducted remotely (online or by phone). Interview duration was between 12 and 83 min with a mean of 47.5 min (one-to-one interviews range = 12–83 min, mean = 46 min; group interviews range = 31–66 min, mean = 51 min).

**Table 1:** Interview participants by setting and role

Setting	Role (& abbreviation for participant IDs for interview quotes)	Participants
***Care Homes*** ^***a***^	*Care Home Managers (CHM)*	*(4)*
	*Deputy Managers (CHDM)*	*(3)*
	*Senior Carer / Carer (SC/JC)*	*(9)*
	***Care Home staff Sub-total***	***16***
	***Care home residents (Res)*** *Age range 87–93; mean age = 90.5yrs*	**3**
	***Family members of care home residents (FM)***	**5**
***Health Service***	*Community nursing staff (CN)*	(6)
	*Digital Care Home Service Team (DCHS)*	(4)
	***Health Service staff Sub-total***	***10***
	***Grand Total***	***34***

**Table 2:** Summary of care homes recruited by provider type, care type and size

Care Home	Participants	Type of Care Home Provider^a^	Care provided^b^	Care home size range (no. of beds)
1	2 x Senior Carers 1 x Carer 1 x Resident	Chain*	Residential	51–60
2	1 x Deputy manager 1 x Senior Carer	Chain*	Residential & Nursing	41–50
3	1 x Deputy manager 1 x Senior Carer	Chain*	Residential & Nursing	51–60
4	1 x Manager	Independent**	Residential	21–30
5	1 x Care Home Manager 1 x Deputy manager 1 x Senior Carer	Chain*	Residential	41–50
6	1 x Care Home Manager	Chain*	Residential	61–70
7	1 x Care Home Manager 2 x Senior Carers	Chain*	Residential	61–70
8	1 x Senior Carer 2 x Residents	Independent**	Residential & Nursing	Up to 20

All care homes had Care Quality Commission rating *‘Good’*

^a^Type of Care Home Provider: Care homes are separate from the National Health Service (NHS) and are largely privatised and run either independently or as part of a larger company or ‘chain’. They are funded through a combination of local authority contributions for eligible individuals, private payments from residents, and NHS support for those with significant healthcare needs [43, 44]

* Homes belonging to a provider organisation with 2 or more care homes in different locations **Homes run as a single organisations

^b^ Care homes vary in the type of care provided. Some have onsite registered nursing staff, (identified here as ‘nursing’ homes). Others (identified here as ‘residential’ homes) offer personal care, such as help with washing and dressing, but no nursing input and rely on external health service providers for residents’ health care needs

Data were drawn from across the range of participant interviews. Two of the five family members interviewed for the wider study had experienced bereavement during the period in question. Therefore, the comments of these two participants are the key ones discussed from the family members’ perspective, illustrating different experiences of losing a relative in a care home at the time. The comments of other family members on topics such as visiting regulations, serve to describe and illustrate some of the experiences of family members at the time.

Three themes were developed from the data, relating to experiences of dying in care homes during the early waves of the COVID-19 pandemic: *(1) Preparing for large scale deaths; (2) Balancing support and policing visiting during the terminal phase*, and *(3) Distress surrounding deaths for staff and families*.

### Preparing for large scale deaths

Interview accounts suggest that at the start of the COVID-19 pandemic, care homes needed to prepare rapidly for uncertain and unprecedented situations. This included making changes in how homes were run, but also included implementation of advance care planning, because it was expected that a large number of residents would become unwell and die.

*“Just before lockdown…we had a nurse came to the home and said to us*,* ‘Right you need to be prepared to hold bodies in the care home. Do you have any cold bedrooms where you can hold bodies?’… and I think that kind of hit us like ‘wow.’ We were thinking*,*…How are we going to cope with this*,* but thankfully that never materialised but we were thinking*,* ‘Oh my god what are we going to do?’”* CHM1.

This sense of anxiety about the unknown appeared to be widespread, with participants reporting a fear that care homes were going to be left to manage on their own. Community nursing teams noted that because of the withdrawal of many other services, care homes were grateful for any support available from their teams.

*“So everybody was fearful and because [we] hadn’t stepped away they were happy to take what we were supporting them with*,* where there was lots of other services that immediately stepped away.” CN1*.

Accounts from care home residents suggest that they were also concerned at the onset of the COVID pandemic, but they generally felt that steps were being taken to protect them from risk.

*Int: “How do you generally feel about this pandemic*,* the coronavirus? What’s your general thoughts on it?*

*Resident2: Well*,* it’s very worrying really. I mean you know*,* it’s obviously very contagious and a lot of people seem to be getting it but you know you just have to take care. I mean the carers are very good in the home. They have their masks on all the time when they’re with you.” (Res2).*

Care home staff described an increase in advance care planning which, in the context of COVID-19 became known as emergency care planning. This included ‘Do not attempt resuscitation’ (DNAR / DNR) orders as well as pre-emptive decisions about whether a resident should be admitted to hospital if they became unwell. Whilst these were generally accepted by care home staff as a necessary step, in some care homes there was concern or mistrust about the function and implications of these, with some reporting feeling worried that residents’ chances of treatment or survival were being set aside. Emergency care planning was largely described as being *done to* the home, rather than initiated within the home, although some care home staff indicated that they were asked to initiate emergency care planning and DNAR discussions themselves which they found difficult and distressing.

*“The community matrons came in around the entire home with residents with DNRs which I didn’t really agree with but… And then they obviously had emergency healthcare plans put in place as well…They spoke to families*,* spoke to residents as well that have their capacity*,* and that was all agreed. At the time it seemed…that they weren’t going to go to hospital*,* if you know what I mean? They were just going to get left to die.” CHM3*.

*“To be honest at the time they weren’t really given much of a choice… We were asked to ring round all of the residents’ families and tell them about it as well. They tried to put that on us as well to do that*,* which wasn’t helpful. Eventually once things calmed down*,* the nurses have started doing that themselves. We just said*,* ‘Look we really don’t want to have this discussion with this person. It’s really going to upset them.”* CHM1.

No family member or resident participants commented on the use of emergency care plans during the early pandemic period, but it appeared from the staff interviews that some attempts were made to speak to them about this.

Care home and health service staff noted that guidelines and policies regarding preparing for deaths occurred within the context of an influx of other frequently changing policies and guidelines, relating to issues such as Personal Protective Equipment (PPE), infection control, and reducing footfall into homes. They reported that in these circumstances, care homes were overwhelmed by information and had limited time to process the information they received, identify what was most pertinent and then implement action. This created a high stress environment for care homes, with death and fear of deaths a central concern.

*“[Care homes] were a bit like everybody really. Goal posts are here one day. Goal posts are there the next day and what I told you yesterday just ignore because it’s now this.” CN1*.

### Balancing support and ‘policing’ visiting during the terminal phase

At the start of the pandemic, visiting in care homes was not allowed, although this rule was later relaxed to allow visits from family members when a resident was dying. Care home staff reflected on the difficulties of managing end-of-life visiting because of restrictions on the length of the visits and who was allowed access. They reported feeling distressed at their conflicting roles; on the one hand they were trying to support residents and families, and on the other they felt required to supervise, impose and enforce social distancing restrictions or to ‘police’ the process.

*“The whole palliative care visits are completely different. You know they only get a set time with their loved ones. It’s not been very nice really… when you’ve got a relative in the room and you’re like*,* ‘I’m sorry your time is up. You’ve got to go.’”* SC7.

This was further compounded by difficulty in identifying when death was imminent, particularly for residents with COVID-19 whose condition could deteriorate rapidly, meaning some family members were unable to visit at all and ‘missed’ seeing their relative at the end of life.

*“One gentleman*,* the family had rang in the morning. I was like*,* ‘Ah yeah he’s absolutely fine*,* no problems.’ By teatime I had to ring them to tell them that he passed away. It was so fast there wasn’t even time for the family to get here to visit.”* CHDM1.

Family members reported that with no ready access to care homes, they relied upon communication from care home staff. The nature of this communication depended on which member of staff was involved in the resident’s care, or indeed which member of staff answered the phone when family called. Therefore, family members reported feeling unsure of what was happening, which was particularly difficult when a resident was nearing the end-of-life.

*“We got absolutely no information out of them whatsoever… you’d try and find out what was happening. It was like a complete inability to get information out of the home. What I later found out*,*- and you find things out later*,* was that there was a massive staff change. A lot of people left*,* obviously they’d had COVID*,* but we didn’t find out. I found out through another resident’s relative who I’d met in the supermarket.”* FM1.

Some family members noted that the limited contact left them unsure of what was going on and mistrustful of the information they were given as they could not see their relative to verify accounts for themselves.

*“Every time we called there was a different update… he wasn’t eating any more*,* he wasn’t drinking*,* he was crying*,* he was very low in mood. They thought this was the end kind of thing. It depended who you spoke to because then the next week they would say he’s drinking a milkshake. It was so confusing*,* we had no idea. It was very quick towards the end. They were saying probably ‘this is it.’ We’d heard that before so we didn’t take it as seriously until they rang to say he’d passed away in the night.”* FM3.

Accounts from some family members suggest that they found the implementation of the restrictions particularly traumatic, especially if their perception was that they were based on rules that appeared to be inconsistent. One family member described their experiences when the care home allowed only the listed next-of-kin to visit when their relative was at the end of life.

*“There was only one person*,*. and when the person was chosen you weren’t allowed to swap so if you wanted to go again it had to be the same person which is the most ridiculous rule.…But he had two children and three grandchildren… They made us choose one person out of five…. [After he died] we were allowed to go and collect his things though which I find even worse. They wanted us to collect all of his stuff and my dad was allowed to go to the care home to do that*,* but he wasn’t allowed to go to visit. So we just found that ridiculous really.”* FM3.

This family member expressed a feeling that the absence of visitors had contributed to their resident relative’s death. They described how their relative had given up, thinking that the family were not going to visit. They also noted that they believed the absence of essential services was also a factor.

*“I think [family being unable to visit] contributed to his death. Although it’s a strong statement I do stand behind it. He stopped eating*,* he stopped drinking and he fell into what we would have at the time if we’d seen him describe as a severe depression. He was crying for us a lot of the time*,* he was asking for us and then he was saying he’s got no family*,* he didn’t remember that he had a family. It was really*,* really distressing but he just declined massively and then the no eating and drinking thing contributed to his kidney failure which ultimately contributed to his death.”* FM3.

Despite experiencing frustration and distress in relation to their interactions with care homes, most family members said that they recognised the difficulties the care homes faced and appreciated efforts made by care home staff to console and accommodate them where possible. Care home staff talked about trying to fill the gap, and attempting to provide comfort when family was unable to be present. Where this was seen to be happening it was gratefully noted by family members who recognised that the staff were trying to provide good care, but said that the lack of visiting especially at the end-of-life was still difficult.

*“Some of them were absolutely exceptional*,* and they really did – some of them really did love her and I know they did. So she got great care but it just wasn’t the same.”* FM4.

There was recognition that care home staff tried to be as flexible and accommodating and considerate as possible with the implementation of restrictions.

*“We had to be all PPE-d up and what have you and… initially I was told I’d be able to spend 10 minutes… half an hour with her*,* but in fact because we were so well known to the staff there and they knew. I think they knew that we weren’t going to go anywhere else or try to do anything*,* I actually was able to stay with her for about two and a half hours… I think the care home did what they could under the circumstances. In fact*,* I think they were probably towards the end more lenient with us than the regulations told them they should be*,* so I mean they were*,* they were brilliant.”* FM4.

We were not able to interview any care home residents who were nearing end-of-life, so were unable to explore the experience of restrictions on end-of-life visiting from residents’ point of view. However, those residents we did speak to appeared to be disappointed but stoical about standard family visiting restrictions at the time.

*“It’s nice to see them. We’re divided by glass*,* you know…I sit on one side and they sit on the other and we talk to each other like that. We can’t touch. We can’t touch each other at all. Oh well it’s not very nice you know*,* – but you know you just have to work with it. There’s nothing you can do about it.” Res2*.

*Int: “What’s that been like? How have you found that sort of change to visiting? What’s that been like for you?”*

*Resident1: “ It hasn’t done much at all. At least I’m seeing them and talking to them and keeping in touch with them and it works very well.” Res1*.

### Distress surrounding deaths for staff and families

Care home staff and family members talked about their distress during the early pandemic period, describing their experiences as ‘horrific’ or ‘awful’. When care homes had a COVID-19 outbreak this could lead to a rapid increase in the number of deaths. Not only did care home staff have to manage their distress about deaths of residents but sometimes also those of colleagues and family members.

*“It was horrific because… it all kind of overlapped. We didn’t have like one*,* and then a break and then one*,* then a break… the ones that we lost went down so fast in the space of hours*.” CHDM1.

Staff were not only distressed by the rate and number of COVID-19 deaths that occurred but the discomfort in which residents were dying, and appeared to be particularly troubled at being unable to relieve residents’ suffering, or provide the end-of-life care that they normally would.

*“This wasn’t standard end-of-life care we would be doing. This was kind of watching people go downhill quite quickly and having so many at the same time in that position was quite distressing because normally when we do end-of-life care we’ve maybe got one or two residents at a time that are in that position.” JC*.

*“Some of our residents had been here for years and they passed away… You know some of our residents with COVID could have been the most placid lovely little old ladies and then they get COVID and they’re just in absolute agony and it was torture to watch them… They were thrashing about the beds*,* they were just really unsettled and screaming out in pain.”* CHM2.

Accounts of health service staff suggest that the losses and distress in care homes were also obvious to these external staff who witnessed them.

*“The care homes that I look after we’ve seen quite a depletion in resident numbers. Massive depletion in resident numbers. One or two of the homes have seen 50% resident demise through COVID and part of that is probably due to the delayed response and then being able to get hold of effective PPE or even how to use PPE.” DCHS2*.

*“It was really upsetting. It was very stressful for everybody because you had carers crying*,* you had relatives crying and we were crying.”* CN6.

Not all care home deaths during the period were due to COVID-19. Staff noted that supporting residents dying from other causes, normally an accepted and expected part of care home life, was also made more difficult due to the restrictions of the pandemic on external visitors, both family and those providing specialist care. Community nursing teams indicated that they were available to assist with the physical nursing aspects of end-of-life care;

*“Yeah but anybody who was end-of-life*,*…we were still going in every day to do with syringe drivers and end of life medication so the carers weren’t left to deal with somebody who was dying… There was always a nurse in there with them.” CN1*.

External support was variable. Care home staff reported that they sometimes felt that their residents needed specialist palliative care services, and the lack of access to these left them feeling unable to manage complex issues that were distressing and beyond their expertise.

*“It was a bit awful because we had a gentleman who came in*,* he was end stages of cancer when he came in and because he came in with the COVID and everything going on we got a phone call from [local hospice] asking how he was and how he settled…and then a week later they said they were taking him off their books because he was here. And [manager] had said*,* ‘Well what about his mental health state? What happens if he needs to talk to somebody and stuff?’ and they were like*,* ‘Well we can’t facilitate it.’…It was a little bit [disappointing] for us because I mean we are carers we do talk to them and stuff but we’re not like mental health teams. We try our best but…”* SC8.

Despite describing their distress in relation to deaths and dying, care home staff also talked about having to get used to it. They appeared to have a strong sense of duty and responsibility to their work and found ways of coping on their own, and through peer support.

*“We’re a good tight bunch aren’t we?…We’re all quite close… I know it sounds silly*,* but you’ve just got to get on with it haven’t you? […] I mean it has been hard. I can’t say it’s been easy but we seem to be used to it now.”* SC2.

Much of the distress experienced around death and dying in care homes was related to the restrictions on end-of-life visits as discussed earlier. Care home staff expressed distress at witnessing families being separated or restricted when residents died, with their comments suggesting moral distress, which may arise when staff are unable to perform their role in a way that is consistent with their values of good care.

*“It’s just an awful position to be in because who are we to say they can’t say their goodbyes and for how long. That’s the bit that I find difficult because I just think it’s awful.” S*C7.

Interview accounts suggest that the distress for families was profound. After an initial acceptance that care homes needed shielding, the ongoing separation, especially at the end-of-life, was reported as very upsetting. Families talked about feeling desperate as they tried to gain access and visit their relative who was dying.

*“When we were told that she was end of care [sic] then we really had to go round and absolutely beg them to go and see her because we were desperate*,* we couldn’t have her just lying there dying without her knowing that we still loved her and cared for her and all that kind of thing. So they did then in the end say that we could all – we were all allowed to go in.”* FM4.

Restrictions around visiting had the potential to cause wider difficulties within the family. For example, Family member 3, whose family had to choose one person to be allowed to visit, noted that;

*“It did cause quite a few issues. I mean we all basically fell out with each other from it. Not in a bad way but we were all kind of upset with each other that we weren’t able to be the person chosen.”* FM3.

The difficulties and distress around restrictions and access to visit their resident relative at end-of-life appeared to have a continued impact following bereavement. Family member 3 reflected;

*“To end his life without having anyone there with him that he knows. That is just a terrible way to go and I don’t think we’ll ever forgive that really. Yeah he did die on his own*,* he did die by himself*… *It’s difficult isn’t it*,* because I can’t say that I’ve lost someone because of the coronavirus pandemic but I feel like I did. He didn’t die of COVID but it restricted our lives to the point where I wasn’t able to get that time back and he missed it with his children*,* grandchildren*,* great grandchildren so I think just the restrictive nature of it has been very difficult.”* FM3.

None of the family members discussed bereavement support. Although residents were not asked directly about death or dying, in order to avoid causing their distress, they were asked about what they thought about the pandemic and its impact of the care home. None of those interviewed discussed the topic of death or dying either generally, or in relation to COVID-19, and none mentioned witnessing the deaths of others.

## Discussion

Data from these interviews capture the challenges of caring for care home residents at the end-of-life during the early COVID-19 pandemic. Interviews with care home and health service staff suggested the uncertainty they were facing as they prepared and managed care at this time. Care homes had reduced access to specialist external services at the end-of-life, leaving staff feeling relatively isolated and left to manage tasks outside their normal roles. This isolation was reflected even more profoundly by family members who struggled to access their relatives, or at times to find out information about them. The pressure on care home staff to support residents and families at the end-of-life was further complicated and compounded by care home staff also having to police end-of-life visits. Unsurprisingly the emotional impact upon all was considerable.

During the early months of the pandemic, care home staff took on the primary role as lead carers, but this was challenging. Support from specialist palliative care services was seldom discussed in these interviews, but when it was, it suggested that access was limited. In research conducted at a similar time, hospice staff described the difficulties in trying to help care homes. [15, 45] Hospices’ lack of access to care homes may have been a result of the ‘infodemic’ experienced across the sector and care home staff struggling to identify key information [45]. Care home managers were trying to identify the most helpful information but given the large amount of information they received, they tried to prioritise often based upon well-known sources or organisations they had a responsibility to, such as the local authority who they found very helpful.

Fears that Do Not Attempt Resuscitation (DNAR) orders were drawn up too broadly are echo concerns raised at the time [16–18]. The care home staff in this study did not take the lead in the initiation of discussions or the recording of these documents. Community nursing teams usually took on this responsibility. Our findings, together with those of other studies [46, 47] suggest a role for increased training and clarity of in advance care planning for care homes, so that these discussions can be held with care home staff with whom residents and families are familiar rather than relying on external staff. Information about upskilling of care home staff in the areas of end-of-life care and advance care planning was absent from the data in this study. Generally, care home staff struggle to find time for training, but an online training programme for care home staff and families about advance care planning during COVID-19 was launched and evaluated during the pandemic [48]. The training demonstrated immediate impact for staff (including health care assistants) who were able to identify when a resident wanted to have this conversation and felt empowered to have an advance care planning conversation or to talk to someone who could [48]. Similarly, families who received the training felt emboldened to discuss advance care planning with the care home staff due to their greater understanding.

Bradshaw et al. [29] suggested that the relationship-centred care critical for good palliative care provision was disrupted during the early pandemic, but that the impact of this disruption was variable depending on care homes’ integration with local health and social care services suggesting inequity of access. Our findings suggest that where care homes were unable to access support from external services such as specialist palliative care teams they found this difficult and felt morally compromised. With limited access to external support from either palliative care teams or primary health care teams, the important collaborative aspects of end-of-life care in care homes described by Handley et al. (2017) [20] may have been further compromised. Our findings reinforce the value of these collaborative approaches to end-of-life care between care homes and health care teams at all times, and ensuring that this continues during pandemics or other emergencies.

In Marcella et al.’s 2015 [21] study, care home staff described as ‘bad deaths’ those characterised by pain and dying alone. Marcella et al. reported that witnessing these ‘bad deaths’ frequently lead to staff experiencing distress and moral angst [21]. Deaths at the height of the pandemic were likely to be perceived as ‘bad’, with little family involvement, barriers to contact and communication [25], and sometimes limited access to symptom management. As is clear from many of our interviews, care home staff found the witnessing of deaths at this time very distressing. In care homes, residents are typically well-known to staff [32] which is likely to increase the distress felt when residents die, and our data suggest that the frequency and rapidity of deaths during the pandemic intensified this sense of distress. Marcella et al. [21] suggested the lack of institutional support for staff on how to manage deaths and grief can have long term impacts. How these experiences will impact care home staff in the long term is unclear, but even a small impact in a sector with many staff vacancies [4, 19] is likely to be significant.

Many of the care home staff in this study expressed discomfort about the care that they were able to provide towards the end-of-life, especially with regard to having to ‘police,’ or enforce restrictions on end-of-life visits, and they reflected that many aspects of end-of-life care at the time did not align with the care they wished to deliver. Many of their descriptions of the care that they were able to give at this time are consistent with the concept of moral distress [24] where staff may feel ‘*compromised as a moral agent in practicing in accordance with accepted professional values and standards*’ *(p59).*

Regarding the impact on families, our findings echo those of other studies where families reported distress at lack of or inconsistent information about their loved ones in care homes [10, 14, 28]. Our findings indicate that when they felt that their loved one had died alone, and without regular contact with family, bereaved family members could experience profound regrets and distress about this some months after their bereavement, especially where they felt that the imposition of restrictions was arbitrary and inconsistent. Families reported being grateful where they felt that care home staff had ‘gone the extra mile’ and been flexible with the rules, to allow them to spend time with their loved ones at the end-of-life. The long-term impact on families is unknown, but some of the difficulties and emotional distress reflected by family members in this study may have implications for complicated and unresolved grief. [10, 28, 49].

### Limitations

An exploration of experiences of death and dying in care homes was not the primary focus of our original study, but our interviews on COVID-19 in care homes yielded rich data on experiences of death and dying, prompting further analysis on this topic. Our study interview schedule for care home and health service staff included questions about resident deaths, but as this was not the project’s key focus, we did not specifically seek out care homes that had experienced multiple deaths, or family members who had been bereaved. Recruitment of care home residents proved particularly challenging, mainly due to COVID-19 restrictions and pressures. This limited the number of resident interviews to three, leaving a gap in our understanding of residents’ experiences. In addition, as noted in the methods section, in order to avoid distress, neither care home residents nor family members were asked directly about death and dying as an interview prompt. However, they were asked about their experiences of care in care homes and about their concerns about the COVID-19 pandemic, and if they mentioned death, dying or end-of-life care, this was followed up with further sensitive questions to explore their experiences and opinions further. In the case of bereaved family members, participants raised the issue of the death of their relative in a care home early in the interview, probably given its central emotional importance in their experience of care homes during the early pandemic. Whilst only two of the five family members interviewed had been bereaved, their accounts of end-of-life experiences in care homes add a richness of description of their lived experience of bereavement during the pandemic. These add valuable further context to the accounts and reflections from care home and health care staff. None of the residents interviewed mentioned fears about death and dying or witnessing the deaths of others, and whilst it is difficult to draw firm conclusions from this, these issues had not had a significant impact on the pandemic experience of these participants so far. Whilst our sample of residents was small, all were considered by care home staff to have sufficient capacity to take part in interviews, and their comments add to our narrative on the impact of the pandemic and visiting restrictions in care homes. It was difficult to recruit family members through care home managers, so a wider recruitment call was used via social media which may have elicited the views of family members who had had a particularly difficult experience of having a relative in a care home during the early pandemic period.

The limitations of conducting research during pandemic restrictions, including recruiting care homes and conducting online interview were ongoing challenges to the study. However, we managed to gather detailed accounts of end-of-life care experiences from both care home staff and families which we believe make a rich addition to those in other studies.

### Implications

Future pandemics should take into account the needs of the dying, their families and care staff to enable visiting, shared decision making and access to palliative care services. These findings indicate the importance of visiting and meaningful contact at the end-of-life, and policy and practice should aim to facilitate this as a priority at all times, as well as in exceptional circumstances such as pandemics. These measures could help reduce the risk of difficult bereavement for families, and moral distress amongst staff.

Our interviews took place during the difficult first phases of the COVID-19 pandemic when memories of experiences were likely to still be vivid. Future research should focus on the longer-term impact on care home staff and bereaved family members to explore any on the lasting emotional impact of these experiences.

## Conclusions

Caring for dying residents in the early waves of the COVID-19 pandemic was very challenging for care home staff. Isolation, lack of specialist support and pressure to take on new roles made this a difficult time. Discomfort over being thrust into the role of policing family visiting was widespread amongst staff, particularly in an end-of-life context. Families struggled to accept deaths where the resident had been largely alone in their final hours. The long-term impact of this distress for care home staff and families is yet to be realised, and planning for future crises should ensure clear policies for provision of end-of-life care and equity of access to external support and services including specialist palliative care.

## Electronic supplementary material

Below is the link to the electronic supplementary material.

Supplementary Material 1

## Data Availability

The datasets generated and/or analysed during the current study are not publicly available due to potentially identifiable confidential details from interviews, but are available from the corresponding author on reasonable request.

## Footnotes

1 Generally, in the UK, the term *care home* is used to refer to residential long-term care facilities. For the purposes of this paper, we use the generic term ‘care home’ to cover both *nursing homes* which offer 24 h onsite registered nursing staff; and *residential homes* which offer personal care, such as help with washing and dressing, but do not have onsite nursing staff and rely on external health service providers for residents’ healthcare needs.

2 The Digital Care Homes Service aims to enhance the processing of non-urgent referrals between care homes and National Health Service providers, by sharing resident health information through a digital app. Through the app care home staff can record; vital signs to generate a ‘National Early Warning Score (NEWS) [36] as well as free-text contextual information. The referral is reviewed and triaged by a clinician who seeks further information from the care home, or triages to an appropriate service provider.

## Abbreviations

CHM: Care Home Manager
CHDM: Care Home Deputy Manager
CN: Community Nurse
DCHS: Digital Care Home service
HRA: Health Research Authority
JC: Junior Carer
SC: Senior Carer
