# Supplementary material for: Dying in residential care homes during the early COVID-19 pandemic: a qualitative interview study
Source: BMC Geriatr. 2025 Feb 24;25:126. doi: 10.1186/s12877-025-05779-y (PMC11854402; doi:10.1186/s12877-025-05779-y)
Supplement: Supplementary file 1 — Supplementary Material 1 [file 12877_2025_5779_MOESM1_ESM.pdf]

Evaluating the Digital Care Service app in care homes in an area of Northern England in the context of the COVID-19 pandemic: qualitative interview study Interview topic guides

[location and organisation names changed in this appendix to protect anonymity]

Pre-interview housekeeping

In advance of the interview participants will have received a copy of the study information sheet and consent form. Due to the COVID-19 pandemic and the need to maintain social distancing E-consent is being taken, where participants provide a simple electronic signature by typing their name as opposed to signing their name with a pen. The consent form will be emailed back to the researchers either before or on the day of interview.

On the day of the interview, prior to commencement of the interview itself the researcher will:

- introduce themselves to the participant as part of the research team;
- thank participant;
- reiterate the purpose of the study and interview;
- explain processes for ensuring anonymity and confidentiality of interview data and that we would like to audio-record the interview;
- explain how interview data will be used;
- confirm whether the interviewee still wishes to participate and, if so, confirm verbal and written consent.

Participants will be provided with the opportunity to ask any questions.

Post-interview housekeeping

At the end of all interviews the interviewee is thanked for their time and contribution, offered the opportunity to ask questions, and reminded that participation is voluntary and they can ask to have their details removed if they change their minds.

Note: *These are not exclusive topic guides, and questions will be omitted or modified depending on the category of interviewee, and as data collection and initial analysis moves on. The semi-structured style of the interviews will also allow for novel questions to be asked should an interviewee bring up an unanticipated topic requiring the researcher to engage in a novel line of questioning.*

List of Topic Guides

Care home staff **2**

NHS staff **5**

Digital Care service staff **7**

Residents **9**

Family members **11**

Care home staff

Rapport / Background

Could you tell me about yourself?

- Name, age

Could you tell me about job/role in the care home?

- Job title; how long have you been in that role; how long have you worked in [care home name]; What is a typical day like for you?

Could you tell me a bit about the care home you work at?

- Type of residents (end of life; dementia); Number of residents; Staff mix; Aligned to a GP surgery?; Entertainment; Visiting
- Group or independent?; Monthly/annual fees?; Council contributions?

Impact of COVID-19

What has the pandemic been like at (name of care home)?

- What changes have occurred?; Have you made any specific changes within the home, e.g. in relation to infection control?
    - What specific changes have you made?
- Cases and deaths of COVID-19;
    - If no cases/deaths – why do you think that is?
    - Where cases/deaths occurred – [offer condolences] - could you talk me through why you think the cases/death occurred?; How have these cases/deaths affected the home?
- when did the home "lockdown";

How have you dealt with visiting?

- Virtual
- Through the window
- Visiting guidelines (update published on 1st December)
    - Communication from the Gov in general?

How have residents responded; how about families?

Personal Protective Equipment (PPE)

- supply; what kind of PPE; how have you found wearing PPE; how have residents responded to staff wearing PPE? How about families?
- **INFECTION CONTROL POLICY?**

How has the home dealt with hospitalisations during the pandemic [Digital Care Service could be mentioned here]?

- and discharges?; how has communication with outside services been?

How about testing residents for COVID-19

- What is the protocol (how often; whom; type of test used?)
- How have the residents responded to being tested?
    - How do you talk with residents about testing?
    - How about those with dementia?
        - Probe around possible distress

How about testing staff for COVID-19

- What is the protocol (how often; whom; type of test used?)
- How have staff responded?

What do you think about the roll out of a COVID-19 vaccine? [added December 2020]

- General view
- Should staff be vaccinated – how could this be managed?
- Should residents be vaccinated – how could this be managed?
- Relatives – should they disclose vaccination status? – how could this be managed?

Have there been any changes to how you provide palliative care?

- Have you had any hospice contact or support?
- Have you changed or updated any palliative care training, for example Six Steps?

How about emergency health care plans?

- Have these been updated due to COVID-19?
- If so, who was involved in this update?
- Have they been updated more than once?

How have you managed the issue of visiting and family contact?

- Early in the pandemic v recent months
- Use of technology
- Role of testing

How do you feel about media reports on the pandemic in care homes?

- What about social media?

Can you tell us about the financial impact of the pandemic on your care home?

- How has the pandemic impacted financially on your staff?
    - E.g. clinically vulnerable?
- How have you covered staff shortages?
- Deaths – loss of LA funding?
- Has the business liability been increased due to COVID? How do you feel about this?

Digital Care Service

Standard Implementation [pre-pandemic]

Moving onto the Digital Care Service app. Let's put the pandemic aside for a moment and talk about Digital Care Service before the pandemic happened.

How long has your care home been using the app?

Do you recall how the app was introduced to [care home name]?

- What was the training like?; who delivered the training; how could this have been improved?

In more normal circumstances (when we are not in a pandemic), how would you use the app?

- How often; in what circumstances, as a routine, or when you are concerned, or both?

Again, without thinking about the pandemic, what do you think about the app?

- Useful; appropriate; could it be improved?
- How has using the app affected your day-to-day workload?
- Do you need to enter the Digital Care Service information in other places, such as other electronic systems or written records?

How has the app influenced/changed decision making?

- Has the system ever failed – either the electronics or the processes?

How about taking vital signs observations specifically?

- How have you found this? How have residents responded to having their vital signs taken by staff? How do you feel about taking clinical measurements in the residents' home?

Prior to the pandemic, had the app changed how you communicate with external health care services?

- GPs, community nurses/matrons, paramedics; Could this be improved?

In previous research we found that care home staff felt they often weren't listened to when contacting external health services. Have you felt that way?

- What impact has Digital Care Service had on this?

Rapid Implementation [during pandemic]

Moving onto the Digital Care Service app.

How long has your care home been aware of the Digital Care Service app?

- How did you become aware of the app?
- What were your initial thoughts about the app? How/why has your view changed?

How long has your care home been using the Digital Care Service the app?

Do you recall how the app was introduced to [care home name]?

- What was the training like?; who delivered the training; could this have been improved?
- How has using the app affected your day-to-day workload?
- Do you need to enter the Digital Care Service information in other places, such as other electronic systems or written records?
- Has the system ever failed – either the electronics or the processes?

Digital Care Service and COVID-19

How have you used Digital Care Service during the pandemic?

- More/less than before; How can it support your work; Does it inhibit your work at all? Why/why not?

How about taking vital signs?

- Are you taking particular measurements? (such as temperature); Are you recording a NEWS2 each time you use the app?

How have you found using NEWS2 during the pandemic?

- Has it been helpful? Why/why not; Has it influenced your decision making?; Has it influenced the decision making of external healthcare professionals?

How about communicating with external services – how has Digital Care Service been useful to you?

- How could this be improved?

What would you say are the residents' reactions towards Digital Care Service have been during the pandemic?

- How have they responded to having their vital signs taken during the pandemic?; How have they reacted to the tablet computer?

How about families?

- Are families aware of Digital Care Service? How have they responded?

With the current need for vigilant sanitation, how are you handling the digital tablet and vital signs equipment?

- Challenges; what is the standard procedure; have there been any particular problems?

Do you have any COVID-19 related policies and procedures?

- How have these changed during the pandemic?
- What sort of information have you had from various bodies e.g. NHS England?

Finally, is there anything I have not asked you about Digital Care Service or the pandemic in care homes that you would like to discuss?

NHS staff

Rapport / Background

Could you tell me about yourself?

- Name; age; job title; how long have you been working in health care?; how long have you worked in this role?

Can you tell me a little bit about your role in relation to care homes?

- What relationship do you have with care homes in the area?; What kinds of support do you offer to care homes?; How has your role changed since the outbreak of the pandemic?

Digital Care Service

Pandemic aside, what do you think about the use of Digital Care Service in care homes?

- Do you think there is enough evidence to support the use of such interventions in care homes? Why/why not?

Prior to the pandemic, what did you think about care home staff using the NEWS2 via the Digital Care Service app?

- Were vital signs being taken correctly? Were care homes calculating a full NEWS2 or only taking certain vital signs?

What about the SBAR format for communicating concerns?

- Has this been useful? Could this be improved?

How has the app influenced/changed decision making?

- Speed; appropriateness; challenges?

Are you aware of any awareness raising among NHS staff about the use of Digital Care Service in care homes?

- How effective was this / Could this have been improved? Are NHS services aware of Digital Care Service?

Did you receive any training on the use of Digital Care Service?

- If not, would it have been beneficial; Could this have been improved?

Did you support training within care homes?

- What do you think about the Digital Care Service training in care homes?

Prior to the pandemic, had Digital Care Service changed the way you communicated with care homes?

- In what way?; Was this positive; could this have been improved?

Can Digital Care Service support person centred care?

- Are there situations where it is inappropriate to use Digital Care Service?
- Can residents decline to have their vital signs taken?
    - Is there a protocol for this?

Overall, has Digital Care Service been helpful to you and to care homes, before the pandemic?

- Why/why not?

COVID-19

Can we discuss the impact of COVID-19 on the care homes you cover?

- Outbreaks?

- Deaths
- Resident's well-being
- Wandering with purpose
- Visiting
- Interaction with others
- Families/visiting
- Staff well-being
- Hygiene and infection control protocol?

How about your work with care homes during the pandemic?

- Early in the pandemic v now
- Any changes?

What do you think about the roll out of a COVID-19 vaccine? [added December 2020]

- General view
- Should care home staff be vaccinated – how could this be managed?
- Should residents be vaccinated – how could this be managed?
- Relatives – should they disclose vaccination status? – how could this be managed?

Digital Care Service and COVID-19

What do you think the Digital Care Service system can offer care homes during the pandemic?

- How can it support your work?; Does it inhibit your work at all? Has it influenced your decision making?

How are you directing care home staff to use Digital Care Service now, in the context of the pandemic?

- Are homes actually using it now, and in the way that you would expect?
- What role do you think NEWS2 plays in this pandemic situation? Monitoring residents for COVID-19? Monitoring for other illnesses?
- Are there any particular differences between nursing and non-nursing homes, in their use of NEWS2 during the pandemic?

Overall, has Digital Care Service been helpful to you and to care homes, during the pandemic?

- Why/why not?

How do you feel about media reports on the pandemic in care homes?

- What about social media?

Is there anything I have not asked you about Digital Care Service or the pandemic in care homes that you would like to discuss?

Digital Care service staff

Rapport / Background

Could you tell me about yourself?

- Name; age; job title; how long have you been working in health care?; how long have you worked in this role?

Digital Care Service

If you had to explain Digital Care Service to a lay member of the public, what would you say?

- Purpose?
- Processes
  - when should it be used;
  - who should it be used by;
  - how is it used (talk me through what care home staff have to do to complete an entry);
  - Can care home staff provide extra contextual data;
  - who receives the data;
  - who can access the data;
  - who makes decisions about triage?

What do you think about the Digital Care Service app?

- What is the evidence to support using Digital Care Service?
- What is the evidence to support using NEWS2 in care homes?

Can Digital Care Service support person centred care?

- Are there situations where it is inappropriate to use Digital Care Service?
- Can residents decline to have their vital signs taken?
  - Is there a protocol for this?

Can you talk me through the implementation of Digital Care Service across your area?

- Who was involved in the development stages? Is there a steering group?
- How do you gain feedback from care homes? How has the implementation involved local health care staff?
- What engagement had you carried out with wider services on how to approach this with care homes?
- What is the training like for care home staff using Digital Care Service?
- What was your training like?

How do you feel the care homes have responded to Digital Care Service?

- Any differences across homes?
  - Resident mix (dementia, end of life)
  - Nursing v residential
  - Staffing?
  - Rapid implementation v standard implementation

Prior to the pandemic, what did you think about care home staff using the <u>NEWS2</u> via the Digital Care Service app?

- Were vital signs being taken correctly? Were care homes calculating a full NEWS2 or only taking certain vital signs?

What about the SBAR format for communicating concerns?

- Has this been useful? Could this be improved?

How has the app influenced/changed decision making?

- Speed; appropriateness; challenges?

Are you aware of any awareness raising among NHS staff about the use of Digital Care Service in care homes?

- How effective was this / Could this have been improved? Are NHS services aware of Digital Care Service?

Digital Care Service and COVID-19

What impact has COVID-19 had on care homes in the area?

- Cases/Deaths; care home lockdowns

What do you think the Digital Care Service system can offer care homes <u>during the pandemic</u>?

- Are homes actually using it now, and in the way that you would expect?
- What role do you think NEWS2 plays in this pandemic situation? Monitoring residents for COVID-19? Monitoring for other illnesses?
- Are there any particular differences between nursing and non-nursing homes, in their use of NEWS2 during the pandemic?

Overall, has Digital Care Service been helpful to care homes and services that support them, during the pandemic?

- Why/why not?

How do you feel about media reports on the pandemic in care homes?

- What about social media?

Is there anything I have not asked you about Digital Care Service in care homes that you would like to discuss?

Residents

Rapport / Background

Could you tell me a bit about yourself?

- Age; how long have you been living at [care home]; family

What is it like living at [care home]

- Staff; rooms; activities; other residents

COVID-19

How do you feel about the Coronavirus outbreak?

- has it changed anything at [care home]?;
- how do you feel about staff wearing face coverings, gloves and aprons?;
- are you concerned about the virus?;
- have you been able to stay in contact with family?
  - How have they been? Concerned?
- have you been following coverage in the news?
  - How has it made you feel?
- has the care home done anything to keep morale up
  - Clap for carers / VE day / etc.
- How have other residents been?
  - Anxious; chaotic; calm; supportive
- How have staff been?
  - Anxious; chaotic; calm; supportive

Digital Care Service

As mentioned in the information sheet your received about this study X months ago / recently [care home] has started taking vital signs observations, such as blood pressure and temperature, on residents when they want to check how a resident is feeling. They have been using a ***hand held computer/tablet*** *to record vital signs and use the device to send information about residents' health to NHS community nursing staff. This helps the care home to communicate with NHS staff, who can make more informed decisions about residents' health care.*

Are you aware of this happening within [care home?]

- If no, rephrase to ensure participant has not misunderstood. If the answer is still no – how would you feel about this happening in [care home]?
- If yes – how do you feel about this?
- Do you recall how you became aware of this happening?
- Have you spoken about this with other residents?
- Have you spoken about this with your family?

Have you had your vital signs [blood pressure; temperature] taken by staff at the home?

- If no, rephrase to ensure participant has not misunderstood. If the answer is still no – ask how they would feel about this.
- If yes – how do you feel about this?
- How often do staff take your vital signs?

- How long have staff being taking your vital signs?
- Do staff ask you whether it is okay to take your vital signs before they do so?

Continue with questioning if the participant is aware of intervention and staff taking vital signs.
As mentioned earlier, when staff take your vital signs are you aware of them using a hand held computer/tablet to note down your results?

- How do you feel about this?

How do you feel about your health information being shared with NHS staff through the hand held computer/tablet

- Any concerns?

Digital Care Service and COVID-19

How do you feel about having your vital signs taken during the coronavirus outbreak?

- Reassured; concerned
- How about other residents? Have they said anything?

In terms of the care you received here, is there anything else [care home] has been doing differently since the outbreak?

Is there anything [care home] could be doing better to support residents like yourself during this pandemic?

Is there anything I have not asked you that you would like to discuss?

Family members

Rapport / Background

Could you tell me a bit about yourself?

- Age; occupation; family

Could you tell me about [family member in care home]?

- Age, health conditions; why did they move into a care home? How long have they been there? [follow-up responses and questions relevant to family member's description of resident's situation]

Could you tell me about [care home]?

- Why was this care home chosen? - Reputation? Financial reasons? Location? CQC scores? Availability of a place?
- In general, how have you found the communication about [family member's] health and well-being?
- [if not already clear] How do you feel about [care home] – good/bad

COVID-19

Just briefly, how do you feel about the coronavirus pandemic? [*keep this brief as it could derail interview yet provides contextual info on how the interviewee has experienced the pandemic as a whole and the forms of information they have been influenced by*]

- How has it affected you? Job; other family members; shielding
- How have you found the media coverage of the pandemic in relation to care homes?
    - What about social media?

How has communication between yourself and [care home] been during the pandemic?

- How has it changed?
- Have you received enough communication?
- Could this have been improved?

How has the pandemic affected visiting [family member in care home]?

- Telephone; virtual; through the window

Digital Care Service

As mentioned in the information sheet your received about this study, X months ago / recently [care home] has started taking vital signs observations, such as blood pressure and temperature, on residents when they want to check whether a resident is feeling unwell. They have been using a hand held computer/tablet to record vital signs and use the device to send information about residents' health to NHS community nursing staff. This helps the care home to communicate with NHS staff, who can make more informed decisions about residents' health care. This is called Digital Care Service.

Were you aware of this happening within [care home] before we contacted you?

- If no, what are your thoughts about this? How do you feel about it? Any concerns?
- If yes, do you recall how you were made aware of this happening?
    - What were your initial thoughts? Good/bad?

    - Did you have any questions about Digital Care Service? Were these answered by [care home]?

Have you / would you spoken/speak about Digital Care Service with [family member]?

- Why/ why not?
- Have they raised any concerns?

Digital Care Service and COVID-19

In the context of the pandemic, how do you feel about care home staff using Digital Care Service?

- Good/bad? Concerns?
- What about taking vital signs?

Finally, is there anything I have not asked you that you would like to discuss?
